# Comparison of Mold Populations in Water-Damaged Homes in Australia and the United States

**Published:** 2017

**Authors:** Rossini Gianni, V Neil, D Lark, L Wymer, S Vesper

**Affiliations:** 1 Southwest Research Institute, San Antonio, TX, USA; 2 Mycotox, Wickham, New South Wales, Australia; 3 Mould Lab, Unit 4/52 Industrial Drive Mayfield East, New South Wales, Australia; 4 United States Environmental Protection Agency, Cincinnati, OH, USA

**Keywords:** Mold, Water-damaged, ERMI, Australia

## Abstract

The goal of this study was to examine whether the Environmental Relative Moldiness Index (ERMI) scale created for United States (U.S.) homes was applicable in the assessment of mold contamination for Australian homes. Settled-dust samples were collected in south-eastern Australian homes (n=76) being investigated for possible water-damage and mold contamination. The 36 ERMI molds were quantified in each sample using mold specific quantitative PCR (MSQPCR) and the ERMI value for each home calculated. These homes were then matched to homes in the U.S. with nearly identical ERMI values and the average log10 concentration of each of the 36 molds statistically compared. Most of the 36 ERMI molds were found in Australian water-damaged homes in comparable concentrations to ERMI-matched U.S. homes. The U.S. ERMI scale might provide reasonable estimates of mold contamination in water-damaged Australian homes.

## Introduction

The prevalence of asthma in Australia is one of the highest for any country with 10.2% of the population or about 2.3 million Australians diagnosed with current asthma. The World Health Organization’s (WHO) review of the scientific literature concluded that indoor mold exposure in damp environments and asthma are linked and recommended that mold exposure be minimized [1]. However, in order to minimize mold exposure, one must be able to accurately quantify mold exposure. The traditional methods used to quantify indoor mold exposures, including short-air samples, visual inspection, beta-glucan or ergosterol measurements, have significant quantitative limitations [1,2]. A more scientifically rigorous approach to quantifying indoor mold contamination was needed.

In order to fill this need, mold specific quantitative PCR (MSQPCR) technology was developed, utilizing unique DNA sequences for each mold [3]. The MSQPCR technology was applied in the U.S. Department of Housing and Urban Development’s (HUD) 2006 American Healthy Homes Survey (AHHS) of a nationally representative 1,083 U.S. homes. After collecting a settled-dust sample from each AHHS home, the MSQPCR technology was used to quantify a panel of 36 molds; the 26 Group 1 molds which are more common in water-damaged homes and the ten Group 2 molds which are common in all homes, even without water damage [2]. The home’s mold contamination level was calculated, as shown in equation 1 (Eq.1).

(1) $$ERMI=\overset{26}{\underset{i=1}{\sum }}{\text{log}}_{10}\left(s_{1i}\right)-\overset{10}{\underset{j=1}{\sum }}{\text{log}}_{10}\left(s_{2j}\right)$$

The sum of the logs of the concentrations of ten Group 2 species (s2) is subtracted from the sum of the logs of the concentrations of the 26 Group 1 species (s1) [3]. This difference is the home’s Environmental Relative Moldiness Index (ERMI) value. (The Group 2 molds are subtracted from the Group 1 molds in order to adjust for differences in cleaning habits, use of opened windows for ventilation, types of outdoor vegetation, etc.) [2]. The ERMI values from the 1083 AHHS homes were assembled from lowest to highest to create the ERMI scale for U.S. homes [3]. The ERMI values range from about −10 to 30 (or rarely higher). The 25% of homes with the greatest mold contamination in the U.S. have ERMI values >5.

The higher the ERMI value in a home, the greater is the risk for occupant asthma based on a review of six epidemiological studies of asthma and ERMI values in U.S. homes [4]. The goal of this current study was to determine the relationship between the concentrations of the 36 ERMI molds in Australian homes with ERMI values >5 compared to ERMI-matched homes in the U.S. in order to determine if the U.S. ERMI scale might be useful in quantifying mold contamination in Australian homes.

## Materials and Methods

**Figure 1:**
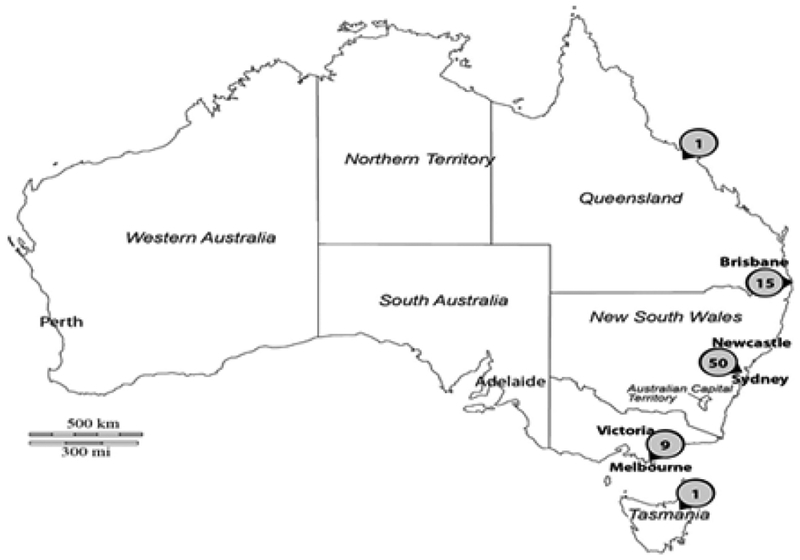
Map of Australian states with the locations and number of the samples from the locations are shown.

Settled-dust samples were collected in the living room and bedroom from southeast Australian (Figure 1) homes/apartments (n=76) that were being investigated for possible mold problems. Then, 5.0 +/− 0.1 mg of sieved-dust were analyzed for the 36 molds in the ERMI panel utilizing each specific MSQPCR assay mix prepared, as previously described [5].

The MSQPCR assay mix contains 12.5 μl of “Universal Master Mix” (Applied Biosystems Inc., Foster City, CA), 1 μl of a mixture of forward and reverse primers at 25 μM each, 2.5 μl of a 400 nM TaqMan probe (Applied Biosystems Inc.), 2.5 μl of 2 mg/ml fraction V bovine serum albumin (Sigma Chemical, St. Louis, MO) and 2.5 μl of DNA free water (Cepheid, Sunnyvale, CA). To this mix was added 5 μl of the DNA extract from the sample. Primers and probes were synthesized commercially (Applied Biosystems, Inc.).

Each Australian home was matched based on its ERMI value to a US home with the closest ERMI value. Ninety percent of the matched, ERMI-value pairs were within ± 0.2 ERMI units of one another. The differences between log10 mean concentrations of each mold species were analyzed using a parametric censored maximum likelihood estimate (MLE) paired t-test via PROC LIFEREG in SAS (v 9.4) for estimation of the potential lower- or upper-censored values of the paired differences [6]. (Non-detects were set at <1 cell per mg dust). Holms-Bonferroni adjustments for multiple comparisons were made to identify significant differences in concentrations of each species based on Chi-square values. Differences with Chi-square values > 10 were significant.

## Results

All of the 36 ERMI molds were detected in Australian homes; attesting to the widespread distribution of these molds. The mean concentration (log10 cells per mg dust) for each of the 36 molds in Australian and U.S. homes with matched ERMI values are presented in Table 1.

Sixteen of the 26 Group 1 molds were found in comparable concentrations in Australian and U.S. ERMI-matched homes. Of the ten Group 1 molds that were different, eight were higher in Australian homes. However, *Aspergillus versicolor* and *Eurotium amstelodami* were more common in U.S. homes. As a result, the average sum of the logs of the Group 1 molds was only 15% greater in Australian compared to matched U.S. homes. It seems likely that the concentrations of the water-damage Group 1 mold were similar in Australian and U.S. homes because, in both countries, homes built since the 1960s have interiors made primarily of plasterboard/drywall. When this building material gets wet, the cellulose covers and organic binding materials make it susceptible to mold growth.

On the other hand, some of the Group 2 molds were probably more common indoors in Australia compared to U.S. homes because residents were more likely to leave the windows open in the milder Australian climate. Still, five of the ten Group 2 molds were found in comparable concentrations in Australian and U.S. ERMI-matched homes (Table 1). *Mucor racemosus* was the only Group 2 mold in significantly greater concentration in U.S. homes. As a result, the average sum of the logs of the Group 2 molds was only 32% greater in Australian compared to U.S. homes.

Because the ERMI value is the result of the of the analysis of a relatively large number of Group 1 and Group 2 molds, small variations for individual species’ concentrations are balanced by the overall ERMI calculation and any differences produced fall within an ERMI value’s standard deviation, i.e., +/− 3 units [2]. Therefore, the application of the MSQPCR technology and the ERMI scale should provide a quantitative estimate of mold contamination in Australian homes comparable to the estimate of mold contamination in U.S. homes.

## Discussion

Most previous studies of mold contamination in Australian homes were based on short-air samples quantified by culturing or based on ergosterol measurements in dust samples [7,8]. Little correlation was seen in estimates of mold contamination which utilized short-air samples taken two years apart in Australian homes [7], emphasizing the limitations of short-air samples in mold analyses [1,2]. In addition, changes in the ergosterol concentrations in dust samples did not correlated with three measures of asthma: asthma in the last 12 months, development of atopy, and development of doctor diagnosed asthma for the home’s occupant [8]. On the other hand, the ERMI scale has been successfully applied to quantifying the association between mold contamination and asthma not only in the U.S. [4] but also in countries as diverse as Scotland [9] and Taiwan [10]. This is the first study to apply the MSQPCR technology and ERMI metric to water–damaged homes in Australia. Of course, there are limitations to the study since it was based on a very small number of homes and only homes in the south-eastern part of Australia (Figure 1). A national sampling of Australian homes, as was done in the U.S. [3] and the creation of an Australian ERMI should improve our ability to quantify mold contamination in Australian homes and any possible links to the high prevalence of asthma in Australia. It is possible that fewer or different molds in an Australian-ERMI might improve the estimates of mold contamination in Australian homes.

## Conclusion

Until an Australian-ERMI is created, it appears that the ERMI value for a water-damaged Australian home is comparable to the ERMI value for a water-damaged U.S. home.

## Figures and Tables

**Table 1:** Comparison of the mean concentration (log10 cells per mg of dust) of each of the 36 Environmental Relative Moldiness Index (ERMI) molds for homes (n=76) in Australia and homes in the United States (U.S.) with matched-ERMI values.

	Mean concentration (log10 cells/mg dust)			Std. Error	95% Confidence Interval		Chi-Square
					From	To	
	Australia	U.S.	M.L.E. Diff.				
*Aspergillus flavus*	0.426	0.96	−0.534	0.269	−1.06	−0.01	3.94
*Aspergillus fumigatus*	1.148	0.723	0.425	0.148	0.14	0.72	8.28
*Aspergillus niger*	1.513	1.061	0.452	0.159	0.14	0.76	8.11
*Aspergillus ochraceus*	0.988	0.277	0.711	0.251	0.22	1.2	8
*Aspergillus penicillioides*	3.132	3.036	0.096	0.157	−0.21	0.4	0.37
*Aspergillus restrictus*	2.064	2.427	−0.363	0.127	−0.61	−0.11	8.14
*Aspergillus sclerotiorum*	1.985	−1.232	3.218	0.473	2.29	4.14	46.25
*Aspergillus sydowii*	0.647	0.742	−0.095	0.277	−0.64	0.45	0.12
*Aspergillus unguis*	1.322	1.554	−0.232	0.333	−0.89	0.42	0.48
*Aspergillus versicolor*	0.012	1.321	−1.309	0.256	−1.81	−0.81	26.15
*Aureobasidium pullulans*	2.091	0.224	1.867	0.266	1.35	2.39	49.38
*Chaetomium globosum*	1.16	0.687	0.474	0.181	0.12	0.83	6.86
*Cladosporium sphaerospermum*	2.535	1.786	0.749	0.169	0.42	1.08	19.64
*Eurotium amstelodami*	2.377	2.84	−0.463	0.144	−0.75	−0.18	10.32
*Paecilomyces variotii*	1.422	1.201	0.221	0.226	−0.22	0.66	0.96
*Penicillium brevicompactum*	1.207	−0.544	1.751	0.443	0.88	2.62	15.61
*Penicillium corylophilum*	1.371	−3.098	4.469	1.161	2.19	6.74	14.82
*Penicillium crustosum*	0.488	0.226	0.262	0.305	−0.34	0.86	0.74
*Penicillium purpurogenum*	0.001	0.768	−0.767	0.393	−1.54	0	3.82
*Penicillium spinulosum*	0.923	1.338	−0.415	0.156	−0.72	−0.11	7.07
*Penicillium variabile*	0.547	0.944	−0.397	0.183	−0.75	−0.04	4.72
*Scopulariopsis brevicaulis*	0.505	1.149	−0.644	0.239	−1.11	−0.18	7.27
*Scopulariopsis chartarum*	0.899	0.306	0.593	0.17	0.26	0.93	12.17
*Stachybotrys chartarum*	0.844	0.896	−0.052	0.293	−0.63	0.52	0.03
*Trichoderma viride*	0.886	0.077	0.809	0.21	0.4	1.22	14.9
*Wallemia sebi*	2.694	2.144	0.55	0.145	0.27	0.84	14.33
Mean Sum Logs Group 1	1.464	1.401	0.062	0.02	0.02	0.1	9.88
*Acremonium strictum*	1.562	1.348	0.214	0.14	−0.06	0.49	2.344
*Alternaria alternata*	0.655	0.801	−0.147	0.19	−0.52	0.22	0.6
*Aspergillus ustus*	0.487	0.654	−0.167	0.186	−0.53	0.2	0.805
*Cladosporium cladosporioides* type 1	2.612	2.686	−0.074	0.114	−0.3	0.15	0.424
*C. cladosporioides* type 2	1.926	0.51	1.416	0.168	1.09	1.75	71.19
*Cladosporium herbarum*	2.555	1.088	1.466	0.166	1.14	1.79	77.96
*Epicoccum nigrum*	3.016	2.036	0.98	0.179	0.63	1.33	29.96
*Mucor racemosus*	0.693	1.711	−1.018	0.153	−1.32	−0.72	44.23
*Penicillium chrysogenum* type 2	1.607	1.069	0.539	0.209	0.13	0.95	6.661
*Rhizopus stolonifer*	0.749	0.068	0.68	0.153	0.38	0.98	19.71
Mean Sum Logs Group 2	1.162	1.041	0.121	0.03	0.06	0.18	16.58

## Future directions

Our goal for the future is to encourage the Australian government to address the relationship between mold contamination and asthma. This would not only include the development of an Australia specific ERMI but also to evaluate the impacts of reducing mold contamination in epidemiological studies of asthma in Australian homes.
